# Rational Water and Nitrogen Management Improves Root Growth, Increases Yield and Maintains Water Use Efficiency of Cotton under Mulch Drip Irrigation

**DOI:** 10.3389/fpls.2017.00912

**Published:** 2017-05-30

**Authors:** Hongzhi Zhang, Aziz Khan, Daniel K. Y. Tan, Honghai Luo

**Affiliations:** ^1^The Key Laboratory of Oasis Eco-agriculture, Xinjiang Production and Construction Group, Shihezi UniversityShihezi, China; ^2^Research Institute of Nuclear and Biological Technologies, Xinjiang Academy of Agricultural SciencesUrumqi, China; ^3^Faculty of Science, Plant Breeding Institute, Sydney Institute of Agriculture, School of Life and Environmental Sciences, The University of Sydney, SydneyNSW, Australia

**Keywords:** cotton yield, irrigation methods, N regime, root architecture, water use efficiency

## Abstract

There is a need to optimize water-nitrogen (N) applications to increase seed cotton yield and water use efficiency (WUE) under a mulch drip irrigation system. This study evaluated the effects of four water regimes [moderate drip irrigation from the third-leaf to the boll-opening stage (W_1_), deficit drip irrigation from the third-leaf to the flowering stage and sufficient drip irrigation thereafter (W_2_), pre-sowing and moderate drip irrigation from the third-leaf to the boll-opening stage (W_3_), pre-sowing and deficit drip irrigation from the third-leaf to the flowering stage and sufficient drip irrigation thereafter (W_4_)] and N fertilizer at a rate of 520 kg ha^-1^ in two dressing ratios [7:3 (N_1_), 2:8 (N_2_)] on cotton root morpho-physiological attributes, yield, WUE and the relationship between root distribution and dry matter production. Previous investigations have shown a strong correlation between root activity and water consumption in the 40–120 cm soil layer. The W_3_ and especially W_4_ treatments significantly increased root length density (RLD), root volume density (RVD), root mass density (RMD), and root activity in the 40–120 cm soil layer. Cotton RLD, RVD, RMD was decreased by 13.1, 13.3, and 20.8%, respectively, in N_2_ compared with N_1_ at 70 days after planting (DAP) in the 0–40 cm soil layer. However, root activity in the 40–120 cm soil layer at 140 DAP was 31.6% higher in N_2_ than that in N_1_. Total RMD, RLD and root activity in the 40–120 cm soil were significantly and positively correlated with shoot dry weight. RLD and root activity in the 40–120 cm soil layer was highest in the W_4_N_2_ treatments. Therefore increased water consumption in the deep soil layers resulted in increased shoot dry weight, seed cotton yield and WUE. Our data can be used to develop a water-N management strategy for optimal cotton yield and high WUE.

## Introduction

Cotton (*Gossypium hirsutum* L.) is a subtropical crop that is mainly grown for natural fiber and oil seed production worldwide (Constable and Bange, 2015). Limitations in soil water levels and low nitrogen (N) concentrations in soil are increasingly becoming a serious problem, especially in arid regions. Soil water and nutrition acquisition is associated with root growth and distribution (Proffitt et al., 1985). To establish a better irrigation system, it is essential to acquire a good understanding of cotton root growth, its spatial distribution in the soil and its relation with yield under different water and N regimes.

Irrigation is crucial to increase crop growth and productivity. However, water supply is often inadequate because of the increased human demand (Wu and Cosgrove, 2000). With increasing concerns over the utilization of scarce water resources, there has been a renewed interest in improving WUE (Tennakoon and Milroy, 2003; Tang et al., 2005). Enhancing irrigation efficiency would improve water management (Hou et al., 2007). Drip irrigation systems are becoming more popular worldwide due to the controlled irrigation rate that fulfills the requirements of a crop during all growth stages (Papadopoulos, 1988; Mmolawa and Or, 2000). Irrigation technology has been improved by linking drip irrigation and plastic film mulching in north-western China (Zhang et al., 2003). Compared with other irrigation methods, a mulch drip irrigation system could conserve 50% of the irrigation water, resulting in a 30% increase in cotton yield. The mulch drip irrigation technology also has the potential for water-conservation and increased yields (Luo et al., 2013). Among the macro nutrients, N is one of the most crucial elements for sustaining crop growth and productivity and its deficiency adversely affects physiological and biochemical processes in plants (Khan et al., 2014, 2017). Nitrogen application to plants subjected to water deficit stress may improve cotton drought tolerance and recovery growth (Zhou and Derrick, 2012). Previous studies have found that application of N fertilizer increase biomass, grain production and WUE in water deficit conditions by increasing the leaf area index (LAI) and sustaining the duration (Latiri-Souki et al., 1998).

Roots play an important role in the uptake of nutrients and water (Hulugalle et al., 2015b), plant hormone production, and organic acid and amino acid synthesis (Yang et al., 2004). Hulugalle et al. (2009) found that cotton root number and root length contributed to soil carbon stocks through intra-seasonal root death. Root morpho-physiological characteristics are positively associated with growth and development (Yang et al., 2008; Chu et al., 2014). Previous research has indicated that fine roots (<2 mm diameter) drive soil processes such as nutrients changes, carbon cycling, sequestration and the activity of soil organisms (Hulugalle et al., 2015a). More roots develop under a drip irrigation system than with furrow irrigation at the early boll filling stage (Hodgson et al., 1990). Wei et al. (2002) reported that mulch drip irrigation systems significantly promote root growth in the upper soil profile. Our previous research found that the water content in the rhizosphere was 50–55% of the WHC prior to drip irrigation and 70–75% of WHC after drip irrigation blocked root growth and decreased root weight (Luo et al., 2010). In summary, under a mulch drip irrigation system, cotton is relatively shallow-rooted, presumably because of the lack of water percolating into the deep subsoil under drip irrigation and the increase in subsoil temperature due to the use of mulch. Yan et al. (2009) reported that root densities of mulch drip irrigation systems may be lower compared with furrow-irrigated and drip irrigated cotton.

The conservation of irrigation water and fertilizer use efficiency are becoming more important in cotton production to achieve optimal yields due to water scarcity and environmental pollution caused by the excessive application of N fertilizer (Haefele et al., 2008). Studies have been conducted on the effects of different water conservation management systems and N fertilization in cotton production (Haefele et al., 2008; Bueno et al., 2010). However, the effects of different water management regimes in combination with different N dressing ratios and the spatial distribution of N in the soil on cotton root growth, yield and WUE are not well documented. Therefore, this study; explored the impact of water and N management regimes on (1) soil water content, root morpho-physiological traits, seed cotton yield and WUE; and (2) determined the relationship between root distribution and dry matter production of a cotton crop.

## Materials and Methods

### Description of Experimental Site

The experiment was conducted on the experimental farm of Shihezi University, Xinjiang, China (45°19′ N, 74°56′ E) in 2009. The average mean temperatures of the 7 months during the growing season (April–October) were 16.1, 12.2, 22.9, 26.2, 22.3, 17.4, and 10.2°C, respectively. The mean rainfall of the 7 months from April to October was 26.1, 46.2, 46.1, 34.0, 33.0, 24.1, and 16.4 mm, respectively. Total evapotranspiration was 1,425 mm during the growing season. The soil at the experimental site was a clay loam with a pH of 7.6, bulk density of 1.41 g m^-3^, organic matter of 11.4 g kg^-1^, available N content of 1.1 g kg^-1^, P_2_O_5_ content of 14.1 mg kg^-1^ and K_2_O content of 182 mg kg^-1^.

### Experimental Design and Field Management

Eight treatment combinations were tested in a split-plot arrangement with four replicates where irrigation was assigned to the main plot and N was applied to the sub-plot (**Table 1**). According to our earlier study (Luo et al., 2013), four irrigation levels were established: (1) moderate drip irrigation (70 ± 5% WHC) from the third leaf to the boll opening stage (W_1_); (2) deficit drip irrigation (55 ± 5% WHC) from the third leaf to the flowering stage and sufficient drip irrigation (80 ± 5% WHC) thereafter (W_2_); (3) pre-sowing irrigation and moderate drip irrigation from the third leaf to the boll opening stage (W_3_); and (4) pre-sowing irrigation and deficit drip irrigation from the third leaf to the flowering stage and sufficient drip irrigation thereafter (W_4_). Nitrogen fertilization in the form of urea (46% N) was applied at the 520 kg ha^-1^ at two different dressing ratios: (1) 7:3 (70% of the N applied at planting, 15% at full budding, and 15% at full flowering: N_1_), 2:8 (20% of the N at planting, 20% at full flowering, 50% at full boll set, and 10% at boll opening: N_2_). The third leaf, full budding stage, full flowering, full boll set, and boll opening stage were 30, 58, 74, 111, and 130 DAP, respectively. Irrigation was supplied using an emitter (release rate = 2.7 L h^-1^) on the top of each soil column every 2 or 3 days after the third leaf had fully expanded.

**Table 1:** Experimental design.

Growth stage	Soil water holding capacity (%)				Proportion of N fertilizer (%)	
	W_1_	W_2_	W_3_	W_4_	N_1_	N_2_
Pre-sowing	–	–	Irrigation	Irrigation	70	20
Three leaf	70	55	70	55	–	–
Full budding	70	55	70	55	15	–
Full flowering	70	55	70	55	15	20
Full boll formation	70	80	70	80	–	50
Boll opening	70	80	70	80	–	10

*W_*3*_ and W_*4*_ were irrigated before sowing. The total amount of N fertilizer was 520 kg ha^-*1*^.*

A cotton cultivar (cv. Xinluzao 43) was grown using polyvinyl chloride (PVC) tubes of 30 cm diameter and 120 cm height. Before sowing, each PVC tube was split vertically into three equal sections. The sections were re-assembled and held in place with a metal sheet. This arrangement allowed the soil and roots to be removed intact from the tubes by separating the three sections. The bottom of each tube was covered with wire mesh to hold the soil. Clay loam soil was taken from the field station, passed through a 2 mm sieve and then packed into the PVC tubes. A pressure compensating drip-irrigation emitter (Beijing Lvyuan Inc., Beijing, China) was fixed in the center of each column. Four cotton seeds were hand sown at 3 cm depth in each column on April 25th. Seeds were placed at a spacing of 10 cm (intra-row × 20 cm inter-row spacing). The soil surface was covered with polyethylene film to reduce evaporation and maintain soil moisture. Four liters of water was supplied to each individual column to enhance the germination rate and seedling development. Weeds and pests were controlled using standard management practices (i.e., application of weedicides and pesticides).

### Data Collection and Observations

#### Soil Moisture and Evapotranspiration

Soil moisture was measured in 10 cm increments between 10 and 100 cm using time domain reflectometry (TDR). The water that was supplied was defined as: A = (*W*_p_ - *W*_a_) ×H, where *A* is the volume of water (mm), *W*_p_ is the field capacity in the 0–40 cm soil layer, *W*_a_ indicates the average relative soil moisture in the 0–40 cm soil layer, and *H* is the thickness of the soil layer. The volume of drip irrigation was managed using a water meter. The consumption ratio of soil water preserved at sowing (R_C_) was assessed according to Luo et al. (2014).

#### Root Distribution and Vigor

Root growth and distribution within each soil columns was determined at 70 and 140 DAP. Six tubes from each treatment were carefully dug out at ground level on each sampling date. The soil columns were cut into 20 cm slices starting from the top of each column. The slices were dipped in water at approximately 60 min and rinsed with tap water. Roots were collected in a 0.5 mm sieve using a water jet. Debris, weeds, and dead roots were separated from ‘live’ roots by hand according to a method used by Gwenzi et al. (2011). There were few dead roots and the live roots were stored in deionized water for further analysis.

Live roots from three of the columns were spread out on a plastic tray contained deionized water and scanned using a flatbed scanner (300 dpi). Root images were analyzed using WinRhizo image analysis software (Regent Instruments, Quebec, QC, Canada). The software was configured to measure root length and root volume. After scanning, the roots were oven-dried at 60°C for 48 h and weighed. The root vigor was measured using the triphenyltetrazolium chloride (TTC) method (Luo et al., 2014).

#### Dry Matter, Yield and WUE

Plants were sampled at ground level and separated into leaves, stems, bolls and seed cotton. Samples were dried to a constant weight at 80°C. Seed cotton was harvested from each column for the determination of total yield. The WUE (kg m^-2^ mm^-1^) for each treatment was calculated by dividing the seed cotton yield m^-2^ by the crop water use. The crop water use was defined as the sum of the total irrigation water and profile water utilized during the whole season.

### Statistical Analysis

Data were analyzed by an analysis of variance (ANOVA) test at a significance level *P* = 0.05 using Statistical Analysis System SPSS v. 11.0 software (SPSS Inc., 1996).

## Results

### Soil Water Content

**FIGURE 1:**
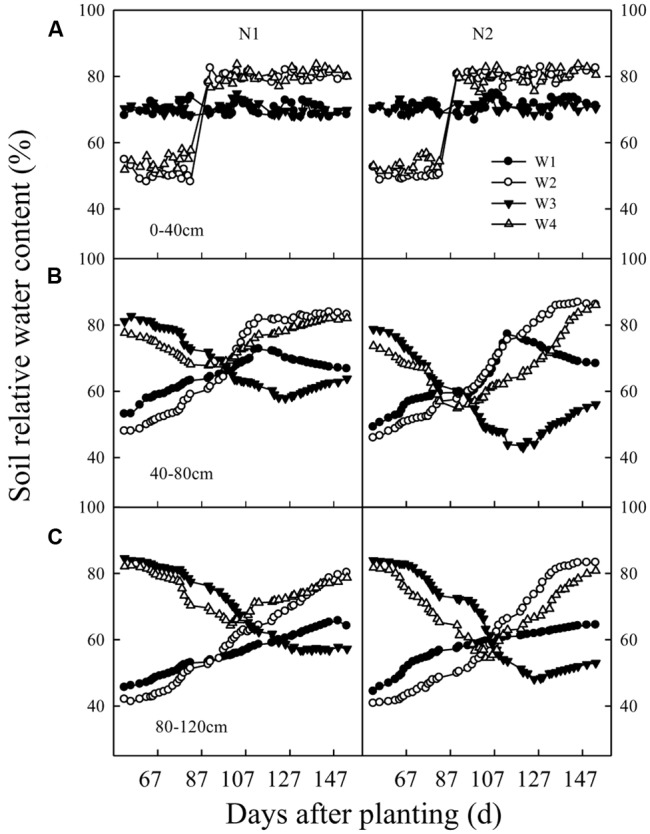
Changes over time in soil relative water content at different depths (**A**: 0–40 cm; **B**: 40–80 cm; and **C**: 80–120 cm) in soil columns as affected by irrigation and N fertilization practice. W_1_, 70 ± 5% water-holding capacity (WHC) from the third-leaf stage to boll-opening; W_2_, 55 ± 5% WHC from the third-leaf stage to flowering and 80 ± 5% WHC thereafter; W_3_, pre-sowing irrigation, 70 ± 5% WHC from the third-leaf stage to boll-opening; W_4_, pre-sowing irrigation, 55 ± 5% WHC from the third-leaf stage to flowering, and 80 ± 5% WHC thereafter; N_1_, 70% of N applied at planting, 15% at full budding, and 15% at full flowering; N_2_, 20% of N at planting, 20% at full flowering, 50% at full boll formation, and 10% at boll opening.

Temporal data regarding the soil moisture content in the 0–40 cm soil layer under the different irrigation water and N fertilizer application regimes are shown in **Figure 1**. The soil moisture content remained at 65–75% WHC in the W_1_ and W_3_ treatments throughout the whole growing season. However, it was higher (75–80%) in the W_2_ and W_4_ at 90 DAP. The soil water content decreased in W_1_ and W_2_ at 65 DAP, although the soil water content in W_3_ from 90 to 150 DAP gradually decreased in the 40–120 cm soil layer.

### Root Growth, Distribution and Activity in Different Soil Profiles

**FIGURE 2:**
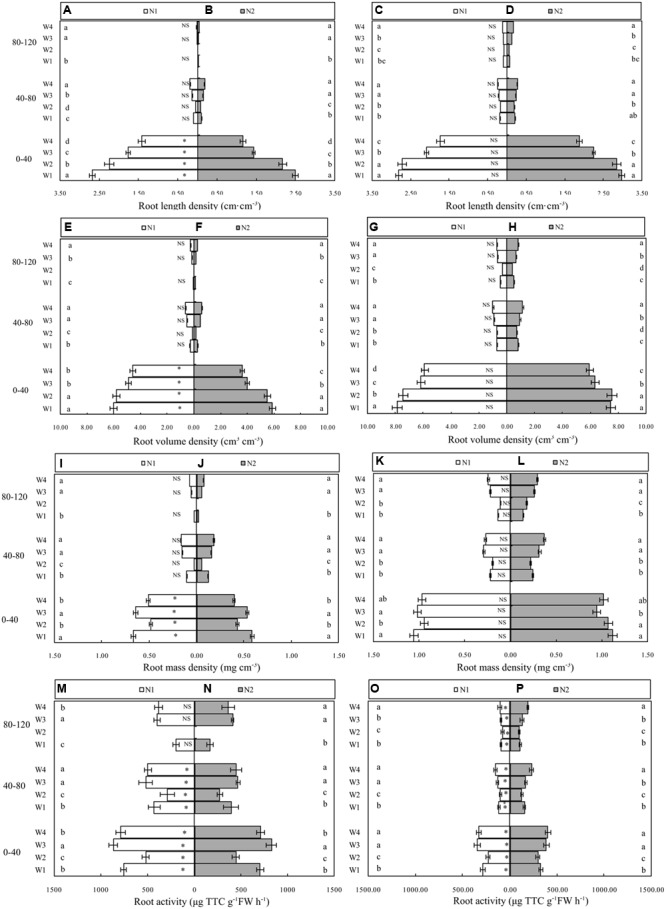
Root length density **(A–D)**, root volume density **(E–H)**, root mass density **(I–L)** and root activity **(M–P)** of cotton 70 and 140 days after planting (Left and Right panels, respectively) as affected by irrigation and N fertilization practice. Soil depth is shown on the far left. Error bars indicate standard error (*n* = 3). Different lowercase letters indicate significant difference (*P* < 0.05) among the four irrigation treatments. ^∗^ and ns indicate significant (*P* < 0.05) and no significant difference (*P* ≥ 0.05), respectively, between N_1_ and N_2_ within an irrigation treatment. W_1_, 70 ± 5% WHC from the third-leaf stage to boll-opening; W_2_, 55 ± 5% WHC from the third-leaf stage to flowering and 80 ± 5% WHC thereafter; W_3_, pre-sowing irrigation, 70 ± 5% WHC from the third-leaf stage to boll-opening; W_4_, pre-sowing irrigation, 55 ± 5% WHC from the third-leaf stage to flowering, and 80 ± 5% WHC thereafter; N_1_, 70% of N applied at planting, 15% at full budding, and 15% at full flowering; N_2_, 20% of N at planting, 20% at full flowering, 50% at full boll formation, and 10% at boll opening. ^∗∗^*P* < 0.01.

Cotton plant root growth, distribution and activity in different soil layers were significantly affected by irrigation water and N application. Cotton RLD increased by 13.0% in N_1_ compared with N_2_ (**Figures 2A,B**). Across the water regimes, RLD progressively increased in W_1_ compared with the other treatments in the 0–40 cm soil layer at 70 DAP under both N_1_ and N_2_. Data regarding RLD under the influence of irrigation water and N at 140 DAP in the 0–120 cm soil layer are presented in **Figures 2C,D**. The RLD was 4.6% greater in N_2_ than N_1_ treatment. Among the irrigation water treatments, W_1_ resulted in a similar RLD to W_2_ under both N_1_ and N_2_, but was significantly higher than that of W_3_ and W_4_, respectively.

No significant differences were observed for cotton RVD under both N regimes in the 0–120 cm soil layer at 70 DAP (**Figures 2E,F**). W_1_ produced a higher RVD in the 0–120 cm soil profile relative to that produced by each of W_2_, W_3,_ and W_4_, respectively. There was significant interaction between N and irrigation water. A higher RVD was obtained in the W_1_N_1_ and W_2_N_1_ combinations than in the W_1_N_2_ and W_2_N_1_ combinations. At the same soil depth, cotton RVD was significantly altered in the later growth stage at 140 DAP. No significant differences were observed between N_1_ and N_2_ in terms of RVD in the 0–120 cm soil layer. However, the W_1_N_1_ and W_2_N_1_ and W_1_N_2_ and W_2_N_1_ combinations resulted in a higher RVD compared with the respective W_3_ and W_4_ combinations (**Figures 2G,H**).

Cotton plant RMD was increased by 15.2% in N_2_ compared with N_1_ in the earlier growth stage (70 DAP). Compared with W_2_ and W_4_, W_1_ and W_3_ produced a higher RMD later in the season (**Figures 2I,J**). Cotton RMD was progressively increased in N_2_ compared with N_1_ at 140 DAP (**Figures 2K,L**). W_3_ and W_4_ were more effective treatments for enhancing cotton RMD than W_1_ and W_2_ under both N_1_ and N_2_.

Temporal data regarding the root activity of cotton at 70 DAP under the influence of N and irrigation water in the 0–120 cm soil layer are presented in **Figures 2M,N**. the root activity of the cotton crop in N_1_ was significantly increased (by 7.3%) compared with N_2_. Across the irrigation treatments, root activity progressively increased in W_3_ and W_4_ compared with W_1_ and W_2_, respectively. Cotton plant root activity was significantly affected by N and irrigation water later in the season (140 DAP). Compared with N_1_ cotton root activity was significantly increased in N_2_ in the 0–120 cm soil layer (**Figures 2O,P**). Compared with W_2_, cotton root activity in W_4_ was significantly increased under both N_1_ and N_2_.

### Relationship between Cotton Root Growth and Soil Moisture

**FIGURE 3:**
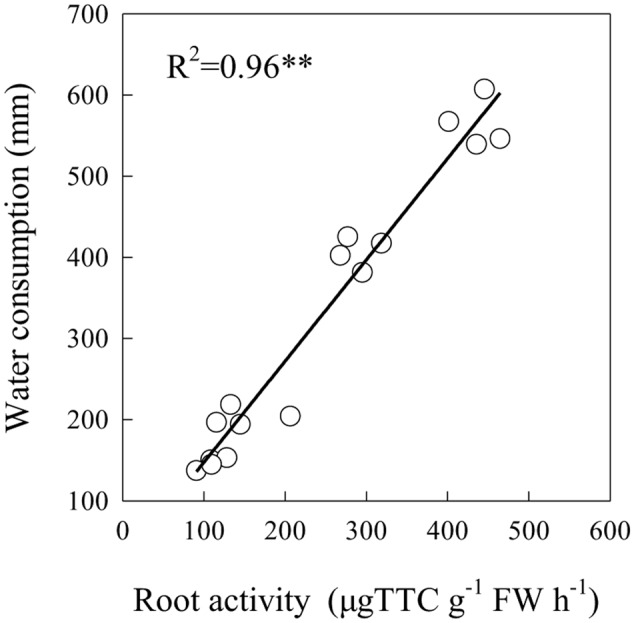
Correlation between cotton root activity in the 40–120 cm soil layer and total water consumption. ^∗∗^*P* < 0.01.

Data regarding the root growth of cotton and its relationship with the soil moisture content are presented in **Figure 3**. Cotton root activity was positively correlated with water consumption at 70 and 140 DAP in the 40–120 cm soil layer. Root activity and soil water consumption in the 40–80 and 80–120 cm layers were significantly higher in W_3_ and W_4_ compared with W_1_ and W_2_ (**Figures 1, 2**). Cotton root activity was higher in the W_4_N_2_ combined treatment than in W_1_N_2_, W_2_N_2_ and W_3_N_2_ combined treatments in the 80–120 cm soil layer.

### Relationship between Cotton Root and Shoot Growth

**FIGURE 4:**
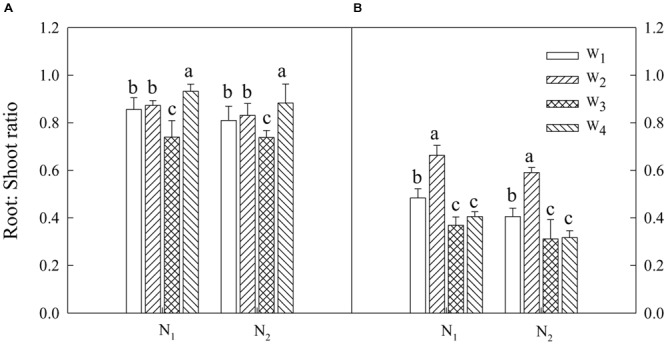
The root: shoot ratio of cotton 70 and 140 days after planting (**A,B**, respectively) as affected by irrigation and N fertilization practice. Error bars indicate standard error (*n* = 3). Bars within an N treatment are significantly different at *P* < 0.05. W_1_, 70 ± 5% WHC from the third-leaf stage to boll-opening; W_2_, 55 ± 5% WHC from the third-leaf stage to flowering and 80 ± 5% WHC thereafter; W_3_, pre-sowing irrigation, 70 ± 5% WHC from the third-leaf stage to boll-opening; W_4_, pre-sowing irrigation, 55 ± 5% WHC from the third-leaf stage to flowering, and 80 ± 5% WHC thereafter; N_1_, 70% of N applied at planting, 15% at full budding, and 15% at full flowering; N_2_, 20% of N at planting, 20% at full flowering, 50% at full boll formation, and 10% at boll opening.

Both irrigation water and N dressing ratios significantly altered the cotton root to shoot ratio at 70 and 140 DAP as shown in **Figure 4**. N_1_ had a higher root to shoot ratio compared with N_2_ during the early growth stage of the crop. Across the irrigation treatments, the root to shoot ratio was significantly higher in W_3_ and W_4_ than in W_1_ and W_2_ (**Figure 4A**). The cotton root to shoot ratio was increased by 18.3% in N_1_ at 140 DAP compared with N_2_ (**Figure 4B**). There was a greater root to shoot ratio in W_3_ than each of W_1_, W_2_, and W_4_.

### Relationship between Cotton Root Growth and Dry Matter Accumulation

**FIGURE 5:**
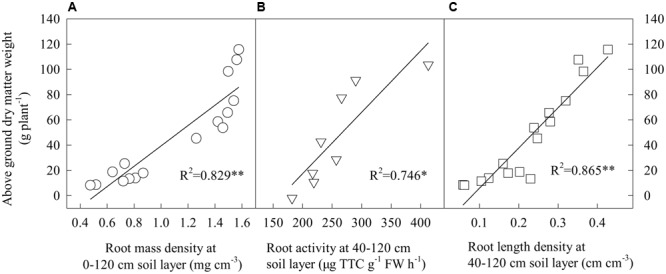
Correlations between cotton shoot dry weight and root growth. ^∗^*P* < 0.05; ^∗∗^*P* < 0.01.

The relationship between root growth and above ground dry matter production was determined in different soil layers (**Figure 5**). Total RMD, RLD and root activity were positively correlated with shoot dry matter production in the 40–120 cm soil layer.

### Water Consumption, Yield and WUE

Data regarding water consumption, yield and WUE under the influence of irrigation water and N regimes are presented in **Table 2**. The total water consumption was greater in N_1_ than N_2_ during the whole growing season. Across the irrigation treatment, the total water consumption was higher in W_4_ than in each of W_1_, W_2_ and W_3_. However, the volume of irrigation water in W_3_ was 5.8 and 10.8% less than in W_1_ and W_2_, respectively. In addition, the volume of water consumed by W_4_ was more than the volume of irrigation water. N_2_ was more effective than N_1_ in the production of both biologic and economic yields. Among the irrigation treatments, the biologic and economic yields for W_3_ and W_4_ were 77 and 50% more than the equivalent yields for W_1_ and W_2_. The WUE of the biologic and economic yields for the W_3_ and W_4_ treatments were progressively increased by 26.0 and 3.6%, 30.5 and 13.1% compared to the equivalent yields for W_1_ and W_2_. In addition, the N_2_ WUE of the biologic and economic yields was 13.4 and 28.8% higher than the equivalent yields of the N_1_ treatment. The W_4_N_2_ combined treatment increased WUE by 28% compared with the W_4_N_1_ combined treatment.

**Table 2:** Cotton water consumption, yield and water use efficiency as affected by irrigation and N practices.

Treatment	Total drip irrigation		Water used from soil		Total water consumption (mm)	Biological yield (kg m^-2^)	Economic yield (kg m^-2^)	WUE of biological yield (kg m^-2^ mm^-1^)	WUE of Economic yield (kg m^-2^ mm^-1^)
	mm	%	mm	%					
W_1_N_1_	476	114.0	–	–	417 de	3038 g	1534 f	7.28 c	3.68 b
W_2_N_1_	504	132.1	–	–	381 e	2566 h	1121 g	6.73 d	2.94 d
W_3_N_1_	460	84.3	86	15.7	546 c	4252 d	2044 d	7.79 c	3.74 b
W_4_N_1_	538	88.7	69	11.3	607 a	5565 c	1789 c	9.17 b	2.95 d
W_1_N_2_	488	114.8	–	–	425 d	3313 f	1563 f	7.79 c	3.78 b
W_2_N_2_	514	127.6	–	–	402 e	3720 e	1308 e	9.25 b	3.25 c
W_3_N_2_	448	83.1	91	16.9	539 c	6088 b	2151 b	11.29 a	3.99 a
W_4_N_2_	527	92.9	41	7.1	567 b	6551 a	2321 a	11.54 a	4.09 a

*Different letters within the same column indicate significant differences at the 0.05 level. W_*1*_, 70 ± 5% water-holding capacity (WHC) from the third leaf to boll opening stage; W_*2*_, 55 ± 5% WHC from the third leaf to flowering stage and 80 ± 5% WHC thereafter; W_*3*_, pre-sowing irrigation, 70 ± 5% WHC from the third leaf to boll opening stage; W_*4*_, pre-sowing irrigation, 55 ± 5% WHC from the third leaf to flowering stage, and 80 ± 5% WHC thereafter; N_*1*_, 70% of N applied at planting, 15% at full budding, and 15% at full flowering stage; N_*2*_, 20% of N at planting, 20% at full flowering, 50% at full boll formation, and 10% at boll opening stage.*

## Discussion

Cotton crops that can endure and recover from drought are needed to minimize yield loss especially in arid regions, and to decrease the water demand of irrigated production. Roots play a significant role in the extraction of water and nutrients from deeper soil layers under water deficit conditions (Luo et al., 2011, 2013; Hassan et al., 2015). A limited supply of water from soil to plants results in a decrease in cooling due to transpiration. This in turn causes an increase in plant temperature (Fuchs, 1990). An inefficient irrigation management leads to a risk that plants will be unable to maintain *in vivo* temperatures at the optimum level for metabolic function and yield losses (Singh et al., 2007). The impact of water deficit stress depends on the severity and duration of water stress and the crop growth stage and genotype (Hassan et al., 2015). Little information has been reported describing root morphology and the physiology of mulch drip irrigation systems and their relationships with cotton yield and WUE under different water and N management. Deficit irrigation results in deeper root penetration of the cotton plant (Sampathkumar et al., 2013), which can conserve 22% of the soil water (Mustafa et al., 2011). Nitrogen application to a cotton crop especially in a water deficit is essential to recover growth and development from drought stress (Khan et al., 2017).

In this study, the W_4_N_2_ combined treatment had a higher root biomass, RLD, root absorption area and root activity than any other treatment. Improvements in the root morphology under W_4_N_2_ contributed to the greater shoot biomass and the optimal cotton yield resulted in an increased WUE. Our data is consistent with the previous literature. Luo et al. (2013) reported that N application and irrigation had significant interactive effects on cotton photosynthesis. Root penetration and proliferation into deeper soil layers could increase soil moisture capture and sustain plant water content especially under water scarce conditions (Luo, 2010; Luo et al., 2011). We found that the W_4_N_2_ combined treatment resulted in a greater root biomass in the 40–120 cm soil layer than the other treatments, with a deeper distribution of roots in the soil, which may contribute to a higher leaf water potential and rate of photosynthesis (Luo et al., 2014). This phenomenon led to an increase in shoot biomass and cotton yield. Our results are consistent with those of Conaty et al. (2015) who showed that a sufficient water supply increased the cotton biomass production at crop maturity.

Root activity has been shown to be an important indicator of the ability of plants to uptake water and nutrients (Liu et al., 2008). The present study identified a very significant correlation between root activity in the 40–120 cm depth and total water consumption. This implies that cotton cultivars with a high level of drought resistance probably have more roots in the middle and deeper soil layers. Previous studies have indicated that water and NO_3_-N rapidly moves from the soil surface to deep layers; therefore, cultivars with deep root systems can rapidly exploit these resources (Lynch, 2013). Liu et al. (2008) suggested that drought stress decreased root activity, while an appropriate N application increased drought resistance in cotton crop. In this study, pre-sowing irrigation and the delayed application of both water and N increased root activity in deep soil layers; thus promoting to absorb water and N from deep layers. Plant root development under moisture stress helps with the sustainable translocation of assimilates to the above ground shoots. Roots are the main source for nutrients supply to shoots; thus, roots and shoots are interdependent on each other. An adjustment in rooting pattern due to a water deficit in the soil will change shoot physiological processes (Hassan et al., 2015). In crop plants with drought resistance, a greater portion of assimilate is allocated toward roots resulting in an increased root to shoot ratio (Riaz et al., 2013; Jamal et al., 2014). However, the impact of a water deficit on the root to shoot ratio can differ from one crop to another, i.e., cotton and maize display a different response under drought stress in terms of the root to shoot ratio (Jamal et al., 2014).

Changes in the root to shoot ratio indicate the demand-supply balance in response to environmental stress (Lambers et al., 1998). Drought stress and nutrient limitation increase carbon translocation from leaves to roots resulting in a greater root to shoot ratio (Andrews et al., 1999; Rahimi et al., 2013). Similarly, we observed that the root to shoot ratio increased as the soil water content decreased at 70 DAP. Limitations in the soil water content can induce carbohydrate accumulation in plant roots (Rahimi et al., 2013). A similar observation was made in the present study, where the root to shoot ratio in water-stressed plants W_2_ and W_4_ was greater than in well-watered plants W_1_ and W_3_ at 140 DAP. These results are consistent with those of Hassan et al. (2015), who also reported that moisture stress significantly increased the cotton root to shoot ratio in a comparison with unstressed plants. In this study, N application under moisture deficit conditions did not significantly influence the root to shoot ratio of cotton. In cotton, a low N application under water deficit conditions can be helpful (Liu et al., 2008). An adequate N application under drought conditions has been shown to improve root growth, but decrease shoot growth (Maranov et al., 1998).

It was found that an inter-dependent relationship existed between the roots and shoot which ensured that adequate supply of carbohydrates to roots could develop and retain active root functions. The resulting high level of root activity improves shoot traits under an adequate supply of nutrients, water and phytohormones to shoots, leading to increased crop productivity (Yang et al., 2004; Chu et al., 2014). We found that root growth was positively related to shoot growth. Thicker root diameters were previously observed in deeper soil layer in high-yielding soybean (Jin et al., 2010). Therefore, we speculated that the improved root morphological and physiological performance for the W_4_N_2_ combined treatment would enhance shoot physiological processes, leading to a higher cotton yield and WUE.

Sufficient water storage in deep soil layers could promote the downward growth of cotton roots, and is essential for achieving water-saving and high-yielding cotton under mulch drip irrigation (Luo et al., 2013, 2014). In the present study, pre-sowing irrigation in W_3_ and W_4_ significantly increased RLD, RVD, RMD, and root activity in 40–120 cm soil layers. This implies that under pre-sowing irrigation and soil water deficit conditions at 70 DAP enhanced root growth into the deeper soil to take up water and nutrients. The N application method should be adjusted according to different irrigation regimes to achieve the maximum yield (Wang et al., 2010). In the present study, RLD, RVD, RMD and root activity in the 0–40 cm soil layer at 70 DAP were significantly lower in N_2_ relative to N_1_. In contrast, root activity in the 40–120 cm soil layer at 140 DAP was significantly higher in N_2_ than in N_1_. These data confirm that the 80 ± 5% WHC combined with 20% of N fertilizer at sowing +80% as topdressing, accelerated root growth and allowed roots to penetrate into the deeper soil layers. Cotton cultivars can access soil water reserves to offset the negative effects of a water deficit during the early growth stages. This allow plants to optimize the distribution of photosynthates between roots and shoots in order to increase dry matter accumulation in shoots with less soil water consumption as well as improving the WUE of the economic yield. Data from the soil column were obtained in the field in order to reflect the actual environmental conditions; however, the conditions were still considerably different than the exact field conditions. Further research is needed to identify the mechanism by which water and N management affects the growth productivity of cotton under a mulch-drip irrigation system.

Maximizing the WUE at all scales is essential in Chinese agricultural systems, especially during drought conditions when water for irrigation is scarce. In this study, the WUE of the biologic and economic yields were maximized in both the W_3_ and W_4_ irrigation regimes. This is probably due to their greater root growth and activity compared to the other regimes. WUE is also associated with daily canopy temperature and WUE was higher when the canopy temperatures were at the optimum of 28.9 ± 1.5°C and 30.8 ± 2.17°C (Conaty et al., 2015). The high WUE in this study might be due to extensive penetration of roots for water extraction and the precise control of N and irrigation water particularly in the mulch drip irrigation system. This data is similar to that of (Conaty et al., 2015), who also reported that good agronomic practices such as fertilizer and water supply can increase WUE. In the future, further research is required to determine the relationship among canopy temperature and cotton yield and WUE under different water and N management regimes especially under water deficit conditions.

## Conclusion

In this experiment irrigation and N regimes significantly altered cotton root growth, root activity, biomass accumulation, WUE and yield. Root activity in the 40–120 cm soil layers depth was positively correlated with water consumption. Shoot dry weight was strongly correlated with RMD, RLD, and root activity. Cotton root growth and activity was increased in the 40–120 cm soil layer under conditions with pre-sowing irrigation and 55% soil WHC from the third-leaf to the flowering stage together with a N fertilizer application of 20% at sowing followed by the remaining 80%. Therefore increased water consumption in the deep soil layers resulted in increased shoot dry weight, seed cotton yield and WUE. Consequently, we suggest decreasing the water and N supply before full flower stage, but increasing the amount of water and N applied at the middle and later growth stages as a high-yield, high-efficiency cultivation technique in the arid area of Xinjiang, China.

## Author Contributions

HL initiated and designed the experiment. HZ performed the experiments and collected the data. HZ and AK analyzed the data and wrote the manuscript. DT, AK, and HL revised the manuscript. All authors read and approved the final manuscript.

## Conflict of Interest Statement

The authors declare that the research was conducted in the absence of any commercial or financial relationships that could be construed as a potential conflict of interest. The reviewer CY and handling Editor declared their shared affiliation, and the handling Editor states that the process nevertheless met the standards of a fair and objective review.

## Abbreviations

DAP: days after planting
RLD: root length density
RMD: root mass density
RVD: root volume density
WHC: water-holding capacity
WUE: water use efficiency.
